# The Genomic Landscape of Melanoma and Its Therapeutic Implications

**DOI:** 10.3390/genes14051021

**Published:** 2023-04-29

**Authors:** Ting-Ting Yang, Sebastian Yu, Chiao-Li Khale Ke, Shih-Tsung Cheng

**Affiliations:** 1 Department of Dermatology, Kaohsiung Medical University Hospital, Kaohsiung Medical University, Kaohsiung 807, Taiwan; 2 Department of Dermatology, Pingtung Hospital, Ministry of Health and Welfare, Pingtung 900, Taiwan; 3 Department of Dermatology, College of Medicine, Kaohsiung Medical University, Kaohsiung 807, Taiwan; 4 Neuroscience Research Center, Kaohsiung Medical University, Kaohsiung 807, Taiwan; 5 Department of Psychiatry, Kaohsiung Medical University Hospital, Kaohsiung 807, Taiwan; 6 Department of Psychiatry, Kaohsiung Municipal SiaoGang Hospital, Kaohsiung Medical University, Kaohsiung 812, Taiwan

**Keywords:** melanoma, gene, mutation

## Abstract

Melanoma is one of the most aggressive malignancies of the skin. The genetic composition of melanoma is complex and varies among different subtypes. With the aid of recent technologies such as next generation sequencing and single-cell sequencing, our understanding of the genomic landscape of melanoma and its tumor microenvironment has become increasingly clear. These advances may provide explanation to the heterogenic treatment outcomes of melanoma patients under current therapeutic guidelines and provide further insights to the development of potential new therapeutic targets. Here, we provide a comprehensive review on the genetics related to melanoma tumorigenesis, metastasis, and prognosis. We also review the genetics affecting the melanoma tumor microenvironment and its relation to tumor progression and treatment.

## 1. Introduction

Melanoma is one of the most aggressive cancer types with an estimated incidence of 325,000 worldwide in the year 2020 and is estimated to continue to grow in the future [1]. Melanoma can be classified into cutaneous melanoma, acral melanoma, mucosal melanoma, and uveal melanoma according to the location of its primary origin. Cutaneous melanoma involving non-glabrous skin is the most common melanoma subtype. It is associated with ultraviolet (UV) radiation exposure and is more common in lighter skin types [1]. Acral melanoma occurs on glabrous skin not commonly exposed to UV radiation and is the most common melanoma subtype observed in patients with darker skin types [2]. Mucosal melanoma is a rare (1.3% of melanomas) but aggressive melanoma subtype originating from melanocytes of mucous membranes [3]. Lastly, uveal melanoma, melanoma arising from the choroid, ciliary body, or iris, represents 5% of melanoma and is more prevalent in populations with lighter skin types [4,5]. The mortality of melanoma remains high, especially for thick lesions and metastatic diseases [6].

The genetic composition of melanoma is complex and varies among different subtypes and individuals, resulting in heterogenous treatment outcomes. Recently, a more in-depth understanding of the genomic landscape of melanoma has been made possible with the aid of next generation sequencing, a sequencing technology making large-scale whole genome sequencing more efficient and practical. Another recent technical advancement is single-cell sequencing, which provides genetic information at the resolution of a single cell. A more thorough understanding of tumor heterogeneity and its microenvironment has been made possible with this technology. The tumor microenvironment, especially immune cells, were shown to profoundly affect survival of melanoma patients [7]. This has become increasingly important as immune checkpoint therapy has become standard treatment for advanced melanoma. However, not all patients respond to immune checkpoint therapy [8]. As our understanding of the complex interaction of tumor cells and the microenvironment increases, opportunities to identify favorable treatment responders have emerged.

In this review, we aim to provide a comprehensive review on the genetic alterations related to melanoma tumorigenesis among different melanoma subtypes, gene mutations related to metastatic melanoma, and the treatment implications of different mutated genes.

## 2. Driver Mutations

Driver mutations occur in the early stages of oncogenesis (i.e., process of normal cells turning into malignant cells) and provide growth advantages to the cancer cells [9]. Identifying these mutations provides potential targets for therapy and information regarding prognosis. Four major genetic subtypes of cutaneous melanoma have been proposed by The Cancer Genome Atlas according to frequently detected hot-spot mutations: mutant *BRAF*, mutant *RAS*, mutant *NF1*, and triple wild-type (TWT), characterized by a lack of *BRAF*, *RAS*, or *NF1* mutations [10]. The most commonly observed *BRAF* and *NRAS* mutations are mutually exclusive [10]. These hot-spot mutations (i.e., mutations observed in significantly increased frequencies compared to normal samples) are all regulators of the mitogen-activated protein kinase (MAPK) pathway involving Ras/RAF/MEK/ERK signaling. Within the TWT subgroup, various driver mutations were observed, including *KIT*, *CTNNB1*, *GNA11*, *GNAQ*, and *EZH2* [10]. Similarly, most driver mutations in the TWT subgroup are MAPK-activating mutations, highlighting the critical role of the MAPK pathway in the pathogenesis of melanoma.

Other frequently involved mutated genes and molecular pathways in the carcinogenesis of melanoma are summarized in Table 1.

### 2.1. Cutaneous Melanomas

Cutaneous melanomas include superficial spreading melanoma (SSM) and lentigo maligna melanoma (LMM). These melanoma subtypes arise from areas of skin exposed to ultraviolet (UV) radiation, with SSM more common on skin with intermittent high-intensity UV radiation while LMM more commonly arises on areas with chronic UV radiation. These subtypes are the most common melanoma subtypes and are more commonly seen in patients with lighter skin types [11]. Due to their relation with UV radiation exposure, they frequently exhibit a high burden of somatic mutation showing high levels of UV radiation signatures, especially C > T substitutions [12]. In comparison, the genetic aberrations in acral and mucosal melanomas are dominated by structural variants [12]. About 50% of UV-related melanomas harbor *BRAF* mutations, with valine (V) to glutamic acid (E) substitution in codon 600 (p.V600E) being the most common mutation [10,12,13,14]. Clinically, patients with BRAF mutations are younger than other subtypes [10,13,14,15]. The second most frequently observed is the *N/K/H-RAS* mutated subtype (20–28%) [10,16]. Contrary to *BRAF*-mutant melanomas, patients with *NRAS*-mutant melanoma tend to be older and have chronic sun UV exposure [17]. Following *RAS* mutation, the third common is the *NF1* mutated subtype (14%) [10]. Lastly, 5–10% are TWT harboring less frequently observed driver mutations [10]. Compared to melanomas with hot-spot mutations, TWT melanomas harbor UV signature mutations less frequently [10]. Different from SSM, LMM was shown to have an increased frequency of non-p.V600E *BRAF*, *NF1*, and *TP53* mutations [15,18].

### 2.2. Acral Melanoma

Acral melanoma, melanomas arising on sun-protected areas such as the palms, soles, and nail units, is the most common melanoma subtype observed in darker skin types [2]. Compared to UV-related cutaneous melanomas, acral melanomas tend to be diagnosed at a more advanced stage and have worse prognosis even after adjusting for Breslow thickness [19]. Acral melanomas share many similar driver mutations with cutaneous melanomas, with *BRAF* (10–34.4%) being the most frequently mutated gene in both Asian and Caucasian cohorts [20,21,22,23]. Most BRAF mutations are a p.V600E substitution and only a few cases are valine (V) to aspartate (D) (p.V600D) substitution or valine (V) to leucine (L) (p.V600L) in codon 600 [15,23,24]. Approximately 9–21.9% acral melanomas have *NRAS* mutations and 11–23% acral melanomas harbor *NF1* mutations [22]. Acral melanomas have a higher proportion of triple-negative melanomas (45–58%) compared to UV-related melanomas [12,15,24]. These triple-negative melanomas most commonly contain *KIT* (10.9–24.4%), GNAQ (18.6%), *TYRP1* mutations [15,20,21,22,23]. Mutations in *TP53*, *PTEN*, *DDX3X*, *RASA2*, *PP6C*, *RAC1*, or *RB1* were less frequently observed in acral melanomas and only reported in limited studies [12,20].

Compared to UV-related melanomas, acral melanomas exhibit a higher frequency of genomic structural variations and lower point mutation burden. Up to 75% of acral melanomas were found to have copy number variations (CNVs), and most AMs exhibit at least one structural variation (SV), including deletions, duplications, tandem duplications, and foldback inversions [12,20,22]. These CNVs and SVs mostly involve cell-cycle aberrations (*CDK4/6, CCND1/2, CDKN2A*) and gains in receptor tyrosine kinase (RTK) related genes and antiapoptosis genes (*BIRC2*, *BIRC3*, *BIRC5*) [15].

### 2.3. Mucosal Melanoma

Other rare melanoma subtypes unrelated to UV radiation include uveal melanoma and mucosal melanoma. Mucosal melanomas (MMs) are more prevalent in Asian populations with a slight female predominance due to increased melanomas involving the female genital tract [25]. Compared to cutaneous melanomas; patients with MM have a significantly lower 5-year survival rate (25%) due to frequent late diagnosis [25,26,27]. *KIT* mutation (19.1–23.1%) was the most frequently found driver mutation in mucosal melanomas followed by *NF1* (7.8–16.4%), *RAS* (6.2–17.9%), and *BRAF* (3.1–16.4%) mutations [27,28,29]. Other significantly mutated genes in MM include *SF3B1* (8.1–11.9%), *SPRED1* (4.0–7.5%), and *ATRX* (6.0%) [28,29]. G-alpha protein *GNAQ/GNA11* has been described to occur in up to 9.5% of MM [30]. Atypical *BRAF* mutations other than the p.V600 hotspot are more frequent in MM [28]. Similar to acral melanoma; MM harbors less mutational burden compared to melanoma associated with UV radiation and exhibits more structural variations and copy number alterations [27,28,29].

### 2.4. Uveal Melanoma

Uveal melanomas (UMs) are melanomas arising from the uveal tract and are the most common tumor of the eye in adults. The overall survival is poor for metastatic UM [4]. Similar to cutaneous melanomas, UMs are more common in patients with lighter skin types [4,31]. However, the genomic landscape for UM is completely different from other melanoma subtypes. UMs typically lack common driver mutations identified in cutaneous melanomas [32]. Significantly mutated driver mutations for UM include *GNAAQ/GNA11* (88–92.5%), *BAP1* (45%), *SF3B1* (24%), *EIEF1AX* (7–17%), *CYSLTR2*, *SRSF2* (4%), *MAPKAPK5*, and *PLCB4* (2.5%) [32,33,34]. These significantly mutated genes had not been identified in CMs [35]. Chromosome copy number aberrations play an important role in risk stratification of UM. Currently, four distinct subtypes related to prognosis are identified based on copy number loss of chromosome 3 and gain of chromosomes 6p and 8q [32].

Table 2 summarizes genetic alteration in different melanoma subtypes.

## 3. Telomere and Melanoma Tumorigenesis

Telomeres are DNA sequences located at the end of chromosomes. Its integrity is crucial in maintaining genome stability and is regulated by telomerase, a ribonucleoprotein enzyme that synthesizes telomeric DNA [43]. Telomerase reverse transcriptase (TERT) aberrations are the most common noncoding mutation (i.e., mutation of regions of DNA that do not code for amino acids) in melanoma expressed in early melanoma progression following MAPK pathway activation [44]. *TERT* promoter is an important regulator of telomerase, whose mutation alters telomere length and promotes tumor cell immortalization [45]. Short telomeres and TERT aberrations are associated with poor survival in melanoma [46,47,48]. In a study including 1019 patients on early melanoma (stage I and II disease) by Rachakonda et al., the hazard ratio for poor melanoma-specific survival was 2.05 (95% CI = 1.33–3.16) for short telomers compared to long telomers [46]. The effect of short telomers on survival was especially prominent in patients under 30 years of age [46]. Patients with TERT promotor mutation and concomitant *BRAF* or *NRAS* mutations were shown to have worse disease-free survival compared to patients with TERT promotor mutation or *BRAF/NRAS* mutations alone [47]. *TERT* mutation can be used to identify melanoma patients with poor prognosis, including relapsing, disease, and death [47]. Over 60% of UV-related melanomas exhibit *TERT* promoter mutations [10]. Within the four major genetic subtypes of cutaneous melanomas, *TERT* promoter mutation is observed in 75% of tumors with *BRAF* mutation but only observed in 6.7% of TWT melanomas [10]. Although less frequent than UV-related melanomas, 9–41% of acral melanomas [21,39] and 30% of mucosal melanomas [29,40] exhibit *TERT* alterations. These are more commonly copy number gains (i.e., gene amplification due to increased gene copies) rather than promoter mutations [40]. Different from other melanoma subtypes, *TERT* alterations are rare in uveal melanomas [41,42].

## 4. Cell Cycle Regulators and Tumor Suppressor Genes

### 4.1. Cell Cycle Regulators

DNA replication in the process of cell proliferation of the human cell is regulated by several cell cycle regulators. These important players in cell regulation include cyclins, cyclin-dependent kinases (CDKs), CDK inhibitors, and the tumor suppressor retinoblastoma (RB) family of proteins [49]. Mutations in genes encoding these regulators may result in aberrant cell proliferation and cancer [49].

As driver mutations occur at the early stages of tumor development, cell cycle regulators play an important role in sustaining cell proliferation in carcinogenesis, resulting in tumor progression. For example, *BRAF* mutation alone is not sufficient for inducing melanoma as *BRAF* mutations are frequently observed in benign nevi [50,51]. Additional genetic alterations in *BRAF*-mutants are required for cancer development [50]. Commonly, aberrations in cell cycle regulation collaborate with driver mutations in the carcinogenesis of melanoma. Significant players in melanoma cell cycle regulation include the *CDKN2A* (cyclin dependent kinase inhibitor 2A) gene which encodes the tumor suppressor p16INK4A, p14ARF, CDKs controlling specific check points of the cell cycle, and the tumor suppressor retinoblastoma protein (RB). Aberrations of the p16INK4A:cyclin D-CK4/6:RB pathway is frequently observed in both cutaneous melanomas and acral melanomas with a prevalence of 68.8–82.7% [10,15,17,52,53]. *CDKN2A* is also the most significant germline mutation with high penetrance in familial melanoma [54].

Mutations of cell cycle regulators *CDKN2A* and *CDKs* were less common in acral melanoma compared with cutaneous melanoma [20]. Melanomas with cell-cycle aberrations are associated with a higher frequency of clinical ulceration, which is associated with worse prognosis [15].

### 4.2. Tumor Suppressor Genes

*TP53* is one of the major tumor suppressor genes (TSGs) regulating cell division and is the most commonly altered gene in human malignancies [55,56]. Its encoded protein p53 plays a critical role in maintaining genomic integrity by promoting cell arrest and apoptosis. Mutations of *TP53* result in rapid tumor progression and metastasis [56]. Despite being the most frequently mutated gene in cutaneous melanoma, *BRAF* hyperactivity alone is not sufficient for melanoma development without concurrent p53 inactivation [57]. Although frequently observed in various human cancers, the incidence of *TP53* mutation (11–30%) is relatively lower in melanoma [58,59], indicating that alterations of other cell cycle regulators, including *CDKN2A* and its products p14ARF and p16INK4A, are more frequently involved in melanoma tumorigenesis [60]. Melanoma patients with *TP53* mutations were shown to have worse prognosis compared to those without the mutation [61,62]. Moreover, *TP53* mutation was shown to be associated with poorer response and poorer overall survival to cytotoxic T-lymphocyte antigen-4 (CTLA-4) blockade immunotherapy in metastatic melanoma due to impaired cytotoxic T-cell-induced apoptosis of tumor cells [61].

Similar to *TP53*, *PTEN* is also a critical tumor suppressor gene in melanoma development. It inhibits cell cycle progression by blocking the PI3K pathway [63]. *PTEN* inactivating mutations occur in the later stages of primary melanoma and are associated with poorer overall survival [63]. *PTEN* loss is also associated with impaired host T cell responses [63,64]. Hence, similar to *TP53* mutation, *PTEN* loss melanomas also show inferior response rate to immune blockade therapies [65,66].

## 5. Metastatic Melanoma

Melanoma is an aggressive cancer and has the tendency to metastasize, leading to the patient’s death [67]. Alterations of the MAPK pathway occur early in cutaneous melanoma tumorigenesis and continue to be amplified in later stages, predominantly by gene duplication as the tumor progresses [44,68]. Currently, there are no genetic mutations exclusively found in metastatic melanoma [44,68,69]. However, several hub genes associated with metastatic melanoma were identified using bioinformatic approaches. These genes include *CDK1*, *BAP1*, *CXCL8*, *THBS1*, *KIT*, *DSG1*, *FLG*, and *PKP1* [70,71,72,73]. Within the identified hub genes, *CDK1* was found to be associated with favorable prognosis [70]. *CDK1* was also associated with increased lymphocyte infiltration, which is also an indicator of favorable prognosis [70]. On the contrary, *DSG1*, *FLG*, and *PKP1* were poor prognostic factors [72]. Loss-of-function mutation of *BAP-1* was associated with poor overall survival [71]. Recently, melanoblast-specific genes, such as *KDELR3* and *KDELR1,* were identified to contribute to the tumor’s ability to metastasize [74]. These findings may provide potential targets for melanoma treatment. Currently, *CDK1* inhibition shows preliminary effects in inducing tumor cell apoptosis in colorectal cancer [75].

## 6. Tumor-Infiltrating Lymphocytes

The behavior of tumor cells is influenced by the surrounding tumor microenvironment, which consists of host immune cells, extracellular matrix, blood vessels, and stroma cells [76]. An important factor influencing melanoma prognosis is the interaction between the host immune cells and tumor cells (i.e., tumor stroma immunobiology). The prognosis of melanoma is more heavily influenced by the tumor stroma immunobiology than the genetic subtypes of driver mutations [10]. Tumor-infiltrating lymphocytes (TIL) have first been shown to be an independent positive prognostic factor for melanoma by Larsen and Grude in 1978 and confirmed by many later studies [77,78,79]. Consequently, as shown in The Cancer Genome Atlas (TCGA) program, advanced cutaneous melanomas expressing genes associated with increased immune cell infiltration and signaling are associated with a favorable survival after adjusting for potential contamination by lymph node tissues [10]. However, it is worth noting that the subclass of lymphocytes also affects prognosis. For example, increased infiltration of regulatory T cells is associated with worse prognosis [80]. Nonetheless, lymphocyte subclass analysis was only performed in limited studies, which may lead to potential biases [80]. Interestingly, in contrast with other melanoma subtypes, increased tumor infiltrating in UM is associated with worse prognosis [32], indicating that UM is a unique subset of melanoma.

## 7. Therapeutic and Prognostic Implications

### 7.1. Targeting the MAPK Pathway

Early studies have shown that *BRAF*-mutant tumors have worse prognosis (median survival 5.7 vs. 8.5 months) in metastatic UV-related melanomas [13]. However, this has not been confirmed by more recent larger studies [10,13,15]. As the most frequently mutated gene in cutaneous melanoma, *BRAF* has become a popular target for melanoma treatment. BRAF inhibitors (BRAFi) are currently approved for *BRAF* V600E and V600K mutant melanoma and successfully improved the progression-free survival and overall survival of patients with these mutations [81,82]. However, the clinical benefits of BRAFi were shown to be short-lived due to drug resistance. The median progression-free survival was 5.1 and 6.8 months for dabrafenib and vemurafenib, respectively [83,84]. MAPK pathway activation by bypassing *BRAF* is the most common mechanism of BRAFi resistance. Hence, by combining *BRAF* and *MEK* (mitogen-activated protein kinase kinase) inhibitors, the survival benefits of these targeted therapies have been prolonged [81,82,85]. Although both *BRAF* V600E and V600K mutant melanoma benefit from BRAFi, melanomas with *BRAF* V600K mutations have shorter progression-free survival under *BRAF* inhibitors but superior response to immune checkpoint inhibitors compared to V600E mutants [10,14,15,18,86]. The progression-free survival for *BRAF* V600E and V600K mutant melanomas was 6.9 and 5.9 months for vemurafenib and 6.3 and 4.5 months in the Phase III trial of dabrafenib [87,88]. Among the different melanoma subtypes, BRAFi benefits cutaneous melanomas the most, as they have the highest frequency of *BRAF* V600 mutations. Mucosal melanoma frequently harbors atypical *BRAF* mutations other than the p.V600 hot spot (such as p.G469A, p.L597Q, p.N581S, p.T599I, and p.G596R), rendering *BRAF* inhibitors that revolutionized melanoma treatment not applicable in this population [28].

NRAS induces cell-cycle dysregulation and cell proliferation by activating downstream signaling of the MAPK pathway [89]. *MEK* inhibitors has been used to treat advanced NRAS-mutant melanoma. However, the results were short-lived, and only resulted in improvement in median progression-free survival for approximately 3 months [90,91,92]. Strategies directly targeting *NRAS* has been attempted by inhibiting farnesyltransferase, a key enzyme in *RAS* maturation, but have not been successfully applied in a clinical setting due to alternative prenylation via geranylgeranyl transferase type 1 [93,94,95,96].

### 7.2. KIT as a Potential Treatment Target

As *KIT* mutations are one of the most frequently mutated genes in melanomas, it is a potential target for advanced melanoma treatment. Although currently there are no approved *KIT* inhibitors for melanoma, *KIT* inhibitor imatinib has shown to provide a durable response to a subset of patients with *KIT* mutant metastatic melanoma with a complete response lasting for more than 1.5 years in a subset of patients [97,98]. Studies on the effeteness of other *KIT* inhibitors including nilotinib and ripretinib are still ongoing [99,100]. *KIT* mutation is relatively rare in uveal melanoma [36]. Nonetheless, most primary uveal melanomas express KIT protein on tumor immunohistochemical analysis, making *KIT* a potential target for treatment. However, the results of *KIT* inhibition by imatinib on UM are disappointing, as no patient showed a response to imatinib in early studies [101,102].

### 7.3. Microphthalmia-Associated Transcription Factor

Microphthalmia-associated transcription factor (MITF) is an important regulator in melanoma development and progression. *MITF* mutation is frequently observed in melanoma [10]. The role of *MITF* in melanoma is paradoxical as it is an oncogene but also has properties in suppressing melanoma invasion and metastasis [103,104]. Compared to melanomas with high levels of *MITF* expression (*MITF*-high), *MITF*-low melanomas are shown to be more invasive [104]. Moreover, melanoma with low *MITF* expression is associated with resistance to *BRAF* inhibition therapy due to reactivation of *ERK* in the MAPK pathway [104]. The expression of *MITF* is negatively correlated with the expression of *AXL*, a receptor tyrosine kinase. *MITF*-low/*AXL*-high melanomas, characterized by low *MITF* expression and high *AXL* markers, are associated with intrinsic resistance to *RAF/MEK* inhibition therapy. Although melanoma can be classified into *MITF*-low/*AXL*-high or *MITF*-high/*AXL*-low melanoma at the tumor level, subclones with *AXL*-high and *MITF*-high expression co-exist in melanoma at the single cell level [105]. The application of BRAFi selectively selects in favor of *AXL*-high subclones and contributes to subsequent drug resistance [105].

### 7.4. TERT Promoter Aberrations

*TERT* promoter mutations are associated with poorer survival and higher metastatic rate in both UV-related and acral melanomas [47,106,107]. Due to the high prevalence of *TERT* promoter aberrations in various melanoma subtypes, it is a potential target for melanoma treatment. As *TERT* alterations are common in *BRAF* V600 mutant melanomas and plays an important role in controlling the apoptosis of cancer cells, combined therapies targeting both *BRAF* and *TERT* may potentially improvement the survival of these patients [108]. Similarly, a high frequency of *TERT* promoter aberrations is also found in *NRAS* mutant melanomas, making *TERT* activity inhibition a potential therapeutic target in this population [109]. *TERT* mutation status has also been used as a potential biomarker indicating favorable response to anti-cytotoxic T lymphocyte-associated antigen 4 (anti-CTLA4) treatment [110].

### 7.5. Cell-Cycle Aberrations

Cell-cycle aberrations are an independent prognostic factor for melanoma-specific survival indicating worse prognosis [15]. As cell-cycle aberrations frequently occur in melanoma, its inhibitors are possible treatment opportunities for these patients. Recently, palbociclib, an FDA-approved *CDK4/6* inhibitor for treating metastatic breast cancer, has been demonstrated to show antitumor effects by inhibiting tumor growth in acral melanoma and mucosal melanoma with *CDK4* amplifications in patient-derived xenograft models [27,52,111]. However, palbociclib only showed a modest effect (20% patients had tumor shrinkage at 8 weeks) as monotherapy in advanced acral melanoma with *CDK4* pathway gene aberrations in a phase II clinical study [112]. Further clinical studies are required to evaluate its effectiveness on melanoma patients. Other *CDK4/6* inhibitors include ribociclib and abemaciclib. Similarly, these agents are currently applied to a limited proportion of melanoma patients and further clinical studies are required to evaluate their effectiveness [113]. Cell-cycle inhibitors combined with targeted therapies against the MAPK pathway have been shown to demonstrate a synergic effect in treating melanoma [111,114,115].

### 7.6. Immune Checkpoint Inhibitors

Immune checkpoint inhibitors (ICIs) targeting programmed cell death protein 1 (PD-1) and cytotoxic T-lymphocyte–associated antigen 4 (CTLA-4) have become the standard care of advanced melanoma in the past 10 years and revolutionized the treatment of melanoma by significantly increasing the overall survival of melanoma patients [116,117,118,119,120]. Not all melanomas respond to ICIs. Currently, only approximately one-third of melanomas respond to ICI treatment [121,122,123]. Within different melanoma subtypes, the response rate to ICIs is lower in acral melanoma compared to cutaneous melanomas. Genetic markers indicating favorable ICI responders in melanoma include high tumor mutational burden [98,124], *BRAF* wild type [98], and increased gene expression of inflammation and T-cell population [10,98,125]. Tumor programmed death-1 ligand 1 (PD-L1) expression is also an important factor influencing therapeutic response to anti-PD-1 immunotherapy, with PD-L1-positive melanoma demonstrating a higher response rate to anti-PD-1 therapy compared to their PD-L1-negative counterpart [126]. Overall, 35% of melanoma express PD-L1, with chronic sun-damaged melanoma showing the highest percentage of PD-L1-positive tumors (62%), followed by mucosal melanoma (44%), acral melanoma (31%), and uveal melanoma (10%) [127]. PD-L1 expression is independent of melanoma driver mutation status (*BRAF/RAS/NF1*) [128]. A more accurate prediction for ICI therapy response may be achieved by combining the above factors.

## 8. Conclusions

The frequencies of key driver gene and pathway alterations differ among different melanoma subtypes, reflecting their heterogenic background in pathogenesis and prognosis. In this era of personal medicine, an in-depth understanding of the genetics of melanoma is crucial in developing personalized treatment and clinical decision-making for targeted therapies of specific genes. Currently, *BRAF*-targeted therapy has been successful in significantly improving overall survival in *BRAF*-mutant melanomas and c-kit inhibitors have been applied in melanomas with the corresponding mutation. Other potential targeted therapies are under development as more significantly mutated genes and pathways have been identified. More recently, ICI therapy has become the standard treatment for advanced melanoma and has significantly improved the survival of these patients. Since only a proportion of patients respond to this revolutionizing therapy, the identification of potential genetic markers indicating favorable responses has become increasingly important. A deepened understanding of the genetic background of melanoma allows us to utilize the best combination of treatments according to the tumor genetic background and predict treatment response more accurately. However, many recently developed targeted therapies are applied only on a limited number of melanoma patients and larger clinical studies are required to verify their effectiveness in this population. It is also worth mentioning that whole genome sequencing is only applied in a limited number of studies and important regulatory noncoding alterations and single-nucleotide variants may be overlooked under whole exome sequencing or low-pass whole genome sequencing.

## Figures and Tables

**Table 1:** Frequently altered pathways and genes in melanoma.

Pathways	Genes Related to Melanoma	Function	Stage of Involvement in Melanoma
MAPK	*BRAF*, *RAS*, *NF1*	Cell cycle proliferation regulation	Early initiation
Telomerase	*TERT* promoter	Gene stabilization	Early initiation
Cell-cycle	*RB1*, *CDKN2A*	Cell cycle regulation	Thin, intermediate melanoma
Apoptosis	*MDM2*, *TP53*	Cell apoptosis	Thick melanoma
PTEN/PI3K/AKT	*PTEN*, *PI3K*	Cell survival and proliferation	Thick melanoma
MITF	*MITF*	Pigmentation, Melanocyte development and differentiation	Thick, advanced melanoma

**Table 2:** Frequency of gene alterations in common melanoma subtypes.

Genes	UV-Related Melanoma	Acral Melanoma	Mucosal Melanoma	Uveal Melanoma
*BRAF*	~50% [10,13,14]	10–34.4% [20,21,22,23]	3.1–16.4% Mutations other than p.v600 codon [27,28,29]	Rare [32,33,34]
*N/H/K-RAS*	20–28% [10,16]	9–21.9% [22]	6.2–17.9% [27,28,29]	Rare [10]
*NF1*	14% [10]	11–23% [22]	7.8–16.4% [27,28,29]	Rare [32,33,34]
*KIT*	1.8% [10]	10.9–24.4% [15,20,21,22,23]	19.1–23.1% [27,28,29]	~9% [36]
*PTEN/PI3K/AKT*	~40–63% [10,37]	Rare [12]	Rare [12]	59% [38]
*TERT*	>60%–86% [10,12]	9–41% [21,39]	30% [29,40]	Rare [41,42]
*GNAAQ/GNA11*	0.9% [10]	18.6% [15,20,21,22,23]	~9.5% [30]	89–92.5% [32,33,34,35]
Other genetic alterations	-	-	*SF3B1* (8.1–11.9%), *SPRED1* (4.0–7.5%), *ATRX* (6%) [28,29]	*BAP1* (45%), *SF3B1* (24%), Most significantly mutated genes are not identified in other melanoma subtypes [32,33,34]

## Data Availability

Not applicable.

## References

[1] Arnold M., Singh D., Laversanne M., Vignat J., Vaccarella S., Meheus F., Cust A.E., de Vries E., Whiteman D.C., Bray F. (2022). Global Burden of Cutaneous Melanoma in 2020 and Projections to 2040. JAMA Dermatol..

[2] Chang J.W.-C. (2013). Acral Melanoma: A Unique Disease in Asia. JAMA Dermatol..

[3] McLaughlin C.C., Wu X.-C., Jemal A., Martin H.J., Roche L.M., Chen V.W. (2005). Incidence of Noncutaneous Melanomas in the U.S. Cancer.

[4] Kaliki S., Shields C.L. (2017). Uveal Melanoma: Relatively Rare but Deadly Cancer. Eye.

[5] Chattopadhyay C., Kim D.W., Gombos D.S., Oba J., Qin Y., Williams M.D., Esmaeli B., Grimm E.A., Wargo J.A., Woodman S.E. (2016). Uveal Melanoma: From Diagnosis to Treatment and the Science in between: Uveal Melanoma Review. Cancer.

[6] Baade P.D., Whiteman D.C., Janda M., Cust A.E., Neale R.E., Smithers B.M., Green A.C., Khosrotehrani K., Mar V., Soyer H.P. (2020). Long-Term Deaths from Melanoma According to Tumor Thickness at Diagnosis. Int. J. Cancer.

[7] Azimi F., Scolyer R.A., Rumcheva P., Moncrieff M., Murali R., McCarthy S.W., Saw R.P., Thompson J.F. (2012). Tumor-Infiltrating Lymphocyte Grade Is an Independent Predictor of Sentinel Lymph Node Status and Survival in Patients with Cutaneous Melanoma. J. Clin. Oncol..

[8] Huang A.C., Zappasodi R. (2022). A Decade of Checkpoint Blockade Immunotherapy in Melanoma: Understanding the Molecular Basis for Immune Sensitivity and Resistance. Nat. Immunol..

[9] Stratton M.R., Campbell P.J., Futreal P.A. (2009). The Cancer Genome. Nature.

[10] Akbani R., Akdemir K.C., Aksoy B.A., Albert M., Ally A., Amin S.B., Arachchi H., Arora A., Auman J.T., Ayala B. (2015). Genomic Classification of Cutaneous Melanoma. Cell.

[11] Whiteman D.C., Pavan W.J., Bastian B.C. (2011). The Melanomas: A Synthesis of Epidemiological, Clinical, Histopathological, Genetic, and Biological Aspects, Supporting Distinct Subtypes, Causal Pathways, and Cells of Origin: The Melanomas. Pigment Cell Melanoma Res..

[12] Hayward N.K., Wilmott J.S., Waddell N., Johansson P.A., Field M.A., Nones K., Patch A.-M., Kakavand H., Alexandrov L.B., Burke H. (2017). Whole-Genome Landscapes of Major Melanoma Subtypes. Nature.

[13] Long G.V., Menzies A.M., Nagrial A.M., Haydu L.E., Hamilton A.L., Mann G.J., Hughes T.M., Thompson J.F., Scolyer R.A., Kefford R.F. (2011). Prognostic and Clinicopathologic Associations of Oncogenic *BRAF* in Metastatic Melanoma. J. Clin. Oncol..

[14] Bauer J., Büttner P., Murali R., Okamoto I., Kolaitis N.A., Landi M.T., Scolyer R.A., Bastian B.C. (2011). BRAF Mutations in Cutaneous Melanoma Are Independently Associated with Age, Anatomic Site of the Primary Tumor, and the Degree of Solar Elastosis at the Primary Tumor Site. Pigment Cell Melanoma Res..

[15] Sheen Y.-S., Tan K.-T., Tse K.-P., Liao Y.-H., Lin M.-H., Chen J.-S., Liau J.-Y., Tseng Y.-J., Lee C.-H., Hong C.-H. (2020). Genetic Alterations in Primary Melanoma in Taiwan. Br. J. Dermatol..

[16] Jakob J.A., Bassett R.L., Ng C.S., Curry J.L., Joseph R.W., Alvarado G.C., Rohlfs M.L., Richard J., Gershenwald J.E., Kim K.B. (2012). NRAS Mutation Status Is an Independent Prognostic Factor in Metastatic Melanoma. Cancer.

[17] Curtin J.A., Fridlyand J., Kageshita T., Patel H.N., Busam K.J., Kutzner H., Cho K.-H., Aiba S., Bröcker E.-B., LeBoit P.E. (2005). Distinct Sets of Genetic Alterations in Melanoma. N. Engl. J. Med..

[18] Sanna A., Harbst K., Johansson I., Christensen G., Lauss M., Mitra S., Rosengren F., Häkkinen J., Vallon-Christersson J., Olsson H. (2020). Tumor Genetic Heterogeneity Analysis of Chronic Sun-damaged Melanoma. Pigment Cell Melanoma Res..

[19] Bradford P.T., Goldstein A.M., McMaster M.L., Tucker M.A. (2009). Acral Lentiginous Melanoma: Incidence and Survival Patterns in the United States, 1986–2005. Arch. Dermatol..

[20] Moon K.R., Choi Y.D., Kim J.M., Jin S., Shin M.-H., Shim H.-J., Lee J.-B., Yun S.J. (2018). Genetic Alterations in Primary Acral Melanoma and Acral Melanocytic Nevus in Korea: Common Mutated Genes Show Distinct Cytomorphological Features. J. Investig. Dermatol..

[21] Zaremba A., Murali R., Jansen P., Möller I., Sucker A., Paschen A., Zimmer L., Livingstone E., Brinker T.J., Hadaschik E. (2019). Clinical and Genetic Analysis of Melanomas Arising in Acral Sites. Eur. J. Cancer.

[22] Newell F., Wilmott J.S., Johansson P.A., Nones K., Addala V., Mukhopadhyay P., Broit N., Amato C.M., Van Gulick R., Kazakoff S.H. (2020). Whole-Genome Sequencing of Acral Melanoma Reveals Genomic Complexity and Diversity. Nat. Commun..

[23] Elefanti L., Zamuner C., Del Fiore P., Stagni C., Pellegrini S., Dall’Olmo L., Fabozzi A., Senetta R., Ribero S., Salmaso R. (2021). The Molecular Landscape of Primary Acral Melanoma: A Multicenter Study of the Italian Melanoma Intergroup (IMI). Int. J. Mol. Sci..

[24] Gao H.-W., Tsai W.-C., Perng C.-L., Wang W.-M., Chiang C.-P. (2018). Distinct MAPK and PI3K Pathway Mutations in Different Melanoma Types in Taiwanese Individuals. Eur. J. Dermatol. EJD.

[25] Patrick R.J., Fenske N.A., Messina J.L. (2007). Primary Mucosal Melanoma. J. Am. Acad. Dermatol..

[26] Hahn H., Lee K., Choi W., Cheong S., Myung K., Hahn H. (2019). An Updated Review of Mucosal Melanoma: Survival Meta-analysis. Mol. Clin. Oncol..

[27] Zhou R., Shi C., Tao W., Li J., Wu J., Han Y., Yang G., Gu Z., Xu S., Wang Y. (2019). Analysis of Mucosal Melanoma Whole-Genome Landscapes Reveals Clinically Relevant Genomic Aberrations. Clin. Cancer Res..

[28] Broit N., Johansson P.A., Rodgers C.B., Walpole S.T., Newell F., Hayward N.K., Pritchard A.L. (2021). Meta-Analysis and Systematic Review of the Genomics of Mucosal Melanoma. Mol. Cancer Res..

[29] Newell F., Kong Y., Wilmott J.S., Johansson P.A., Ferguson P.M., Cui C., Li Z., Kazakoff S.H., Burke H., Dodds T.J. (2019). Whole-Genome Landscape of Mucosal Melanoma Reveals Diverse Drivers and Therapeutic Targets. Nat. Commun..

[30] Sheng X., Kong Y., Li Y., Zhang Q., Si L., Cui C., Chi Z., Tang B., Mao L., Lian B. (2016). GNAQ and GNA11 Mutations Occur in 9.5% of Mucosal Melanoma and Are Associated with Poor Prognosis. Eur. J. Cancer.

[31] Weis E. (2006). The Association between Host Susceptibility Factors and Uveal Melanoma: A Meta-Analysis. Arch. Ophthalmol..

[32] Robertson A.G., Shih J., Yau C., Gibb E.A., Oba J., Mungall K.L., Hess J.M., Uzunangelov V., Walter V., Danilova L. (2017). Integrative Analysis Identifies Four Molecular and Clinical Subsets in Uveal Melanoma. Cancer Cell.

[33] Decatur C.L., Ong E., Garg N., Anbunathan H., Bowcock A.M., Field M.G., Harbour J.W. (2016). Driver Mutations in Uveal Melanoma: Associations with Gene Expression Profile and Patient Outcomes. JAMA Ophthalmol..

[34] Johansson P.A., Brooks K., Newell F., Palmer J.M., Wilmott J.S., Pritchard A.L., Broit N., Wood S., Carlino M.S., Leonard C. (2020). Whole Genome Landscapes of Uveal Melanoma Show an Ultraviolet Radiation Signature in Iris Tumours. Nat. Commun..

[35] Johnson D.B., Roszik J., Shoushtari A.N., Eroglu Z., Balko J.M., Higham C., Puzanov I., Patel S.P., Sosman J.A., Woodman S.E. (2016). Comparative Analysis of the *GNAQ*, *GNA11*, *SF3B1*, and *EIF1AX* Driver Mutations in Melanoma and across the Cancer Spectrum. Pigment Cell Melanoma Res..

[36] Wallander M.L., Layfield L.J., Emerson L.L., Mamalis N., Davis D., Tripp S.R., Holden J.A. (2011). KIT Mutations in Ocular Melanoma: Frequency and Anatomic Distribution. Mod. Pathol..

[37] Tsao H., Mihm M.C., Sheehan C. (2003). PTEN Expression in Normal Skin, Acquired Melanocytic Nevi, and Cutaneous Melanoma. J. Am. Acad. Dermatol..

[38] Li Y., Shi J., Yang J., Ge S., Zhang J., Jia R., Fan X. (2020). Uveal Melanoma: Progress in Molecular Biology and Therapeutics. Ther. Adv. Med. Oncol..

[39] Rabbie R., Ferguson P., Molina-Aguilar C., Adams D.J., Robles-Espinoza C.D. (2019). Melanoma Subtypes: Genomic Profiles, Prognostic Molecular Markers and Therapeutic Possibilities. J. Pathol..

[40] Ma Y., Xia R., Ma X., Judson-Torres R.L., Zeng H. (2021). Mucosal Melanoma: Pathological Evolution, Pathway Dependency and Targeted Therapy. Front. Oncol..

[41] Griewank K.G., Murali R., Schilling B., Scholz S., Sucker A., Song M., Süsskind D., Grabellus F., Zimmer L., Hillen U. (2013). TERT Promoter Mutations in Ocular Melanoma Distinguish between Conjunctival and Uveal Tumours. Br. J. Cancer.

[42] Dono M., Angelini G., Cecconi M., Amaro A., Esposito A.I., Mirisola V., Maric I., Lanza F., Nasciuti F., Viaggi S. (2014). Mutation Frequencies of GNAQ, GNA11, BAP1, SF3B1, EIF1AX and TERT in Uveal Melanoma: Detection of an Activating Mutation in the TERT Gene Promoter in a Single Case of Uveal Melanoma. Br. J. Cancer.

[43] Maciejowski J., de Lange T. (2017). Telomeres in Cancer: Tumour Suppression and Genome Instability. Nat. Rev. Mol. Cell Biol..

[44] Shain A.H., Joseph N.M., Yu R., Benhamida J., Liu S., Prow T., Ruben B., North J., Pincus L., Yeh I. (2018). Genomic and Transcriptomic Analysis Reveals Incremental Disruption of Key Signaling Pathways during Melanoma Evolution. Cancer Cell.

[45] Chiba K., Johnson J.Z., Vogan J.M., Wagner T., Boyle J.M., Hockemeyer D. (2015). Cancer-Associated TERT Promoter Mutations Abrogate Telomerase Silencing. eLife.

[46] Rachakonda S., Srinivas N., Mahmoudpour S.H., Garcia-Casado Z., Requena C., Traves V., Soriano V., Cardelli M., Pjanova D., Molven A. (2018). Telomere Length and Survival in Primary Cutaneous Melanoma Patients. Sci. Rep..

[47] Nagore E., Heidenreich B., Rachakonda S., Garcia-Casado Z., Requena C., Soriano V., Frank C., Traves V., Quecedo E., Sanjuan-Gimenez J. (2016). *TERT* Promoter Mutations in Melanoma Survival: *TERT* Promoter Mutations in Melanoma Survival. Int. J. Cancer.

[48] Griewank K.G., Murali R., Puig-Butille J.A., Schilling B., Livingstone E., Potrony M., Carrera C., Schimming T., Möller I., Schwamborn M. (2014). TERT Promoter Mutation Status as an Independent Prognostic Factor in Cutaneous Melanoma. JNCI J. Natl. Cancer Inst..

[49] Leal-Esteban L.C., Fajas L. (2020). Cell Cycle Regulators in Cancer Cell Metabolism. Biochim. Biophys. Acta BBA Mol. Basis Dis..

[50] Paluncic J., Kovacevic Z., Jansson P.J., Kalinowski D., Merlot A.M., Huang M.L.-H., Lok H.C., Sahni S., Lane D.J.R., Richardson D.R. (2016). Roads to Melanoma: Key Pathways and Emerging Players in Melanoma Progression and Oncogenic Signaling. Biochim. Biophys. Acta BBA Mol. Cell Res..

[51] Pollock P.M., Harper U.L., Hansen K.S., Yudt L.M., Stark M., Robbins C.M., Moses T.Y., Hostetter G., Wagner U., Kakareka J. (2003). High Frequency of BRAF Mutations in Nevi. Nat. Genet..

[52] Kong Y., Sheng X., Wu X., Yan J., Ma M., Yu J., Si L., Chi Z., Cui C., Dai J. (2017). Frequent Genetic Aberrations in the CDK4 Pathway in Acral Melanoma Indicate the Potential for CDK4/6 Inhibitors in Targeted Therapy. Clin. Cancer Res..

[53] Young R.J., Waldeck K., Martin C., Foo J.H., Cameron D.P., Kirby L., Do H., Mitchell C., Cullinane C., Liu W. (2014). Loss of *CDKN2A* Expression Is a Frequent Event in Primary Invasive Melanoma and Correlates with Sensitivity to the CDK4/6 Inhibitor PD0332991 in Melanoma Cell Lines. Pigment Cell Melanoma Res..

[54] Zocchi L., Lontano A., Merli M., Dika E., Nagore E., Quaglino P., Puig S., Ribero S. (2021). Familial Melanoma and Susceptibility Genes: A Review of the Most Common Clinical and Dermoscopic Phenotypic Aspect, Associated Malignancies and Practical Tips for Management. J. Clin. Med..

[55] Hollstein M., Sidransky D., Vogelstein B., Harris C.C. (1991). P53 Mutations in Human Cancers. Science.

[56] Kastenhuber E.R., Lowe S.W. (2017). Putting P53 in Context. Cell.

[57] Patton E.E., Widlund H.R., Kutok J.L., Kopani K.R., Amatruda J.F., Murphey R.D., Berghmans S., Mayhall E.A., Traver D., Fletcher C.D.M. (2005). BRAF Mutations Are Sufficient to Promote Nevi Formation and Cooperate with P53 in the Genesis of Melanoma. Curr. Biol. CB.

[58] Weiss J., Heine M., Arden K.C., Körner B., Pilch H., Herbst R.A., Jung E.G., Garbe C., Schmitz S., Orfanos C.E. (1995). Mutation and Expression of TP53 in Malignant Melanomas. Skin Cancer: Basic Science, Clinical Research and Treatment.

[59] Hussein M.R. (2004). The *TP53* Tumor Suppressor Gene and Melanoma Tumorigenesis: Is There a Relationship?. Tumor Biol..

[60] Hocker T.L., Singh M.K., Tsao H. (2008). Melanoma Genetics and Therapeutic Approaches in the 21st Century: Moving from the Benchside to the Bedside. J. Investig. Dermatol..

[61] Xiao W., Du N., Huang T., Guo J., Mo X., Yuan T., Chen Y., Ye T., Xu C., Wang W. (2018). TP53 Mutation as Potential Negative Predictor for Response of Anti-CTLA-4 Therapy in Metastatic Melanoma. EBioMedicine.

[62] Gould Rothberg B.E., Berger A.J., Molinaro A.M., Subtil A., Krauthammer M.O., Camp R.L., Bradley W.R., Ariyan S., Kluger H.M., Rimm D.L. (2009). Melanoma Prognostic Model Using Tissue Microarrays and Genetic Algorithms. J. Clin. Oncol. Off. J. Am. Soc. Clin. Oncol..

[63] Cabrita R., Mitra S., Sanna A., Ekedahl H., Lövgren K., Olsson H., Ingvar C., Isaksson K., Lauss M., Carneiro A. (2020). The Role of PTEN Loss in Immune Escape, Melanoma Prognosis and Therapy Response. Cancers.

[64] Dong Y., Richards J.-A., Gupta R., Aung P.P., Emley A., Kluger Y., Dogra S.K., Mahalingam M., Wajapeyee N. (2014). PTEN Functions as a Melanoma Tumor Suppressor by Promoting Host Immune Response. Oncogene.

[65] Peng W., Chen J.Q., Liu C., Malu S., Creasy C., Tetzlaff M.T., Xu C., McKenzie J.A., Zhang C., Liang X. (2016). Loss of PTEN Promotes Resistance to T Cell–Mediated Immunotherapy. Cancer Discov..

[66] Vidotto T., Melo C.M., Castelli E., Koti M., dos Reis R.B., Squire J.A. (2020). Emerging Role of PTEN Loss in Evasion of the Immune Response to Tumours. Br. J. Cancer.

[67] Braeuer R.R., Watson I.R., Wu C.-J., Mobley A.K., Kamiya T., Shoshan E., Bar-Eli M. (2014). Why Is Melanoma So Metastatic?. Pigment Cell Melanoma Res..

[68] Vergara I.A., Mintoff C.P., Sandhu S., McIntosh L., Young R.J., Wong S.Q., Colebatch A., Cameron D.L., Kwon J.L., Wolfe R. (2021). Evolution of Late-Stage Metastatic Melanoma Is Dominated by Aneuploidy and Whole Genome Doubling. Nat. Commun..

[69] Manca A., Paliogiannis P., Colombino M., Casula M., Lissia A., Botti G., Caracò C., Ascierto P.A., Sini M.C., Palomba G. (2019). Mutational Concordance between Primary and Metastatic Melanoma: A Next-Generation Sequencing Approach. J. Transl. Med..

[70] Luan H., Jian L., He Y., Zhang T., Zhou L. (2022). Exploration and Validation of Metastasis-Associated Genes for Skin Cutaneous Melanoma. Sci. Rep..

[71] Chen J., Wu F., Shi Y., Yang D., Xu M., Lai Y., Liu Y. (2019). Identification of Key Candidate Genes Involved in Melanoma Metastasis. Mol. Med. Rep..

[72] Jia C.-L., Yang F., Li R.-N. (2022). Identification of Potential Core Genes Between Primary and Metastatic Malignant Melanoma and Analysis of Their Immune Correlation. Int. J. Gen. Med..

[73] Su W., Guan Y., Huang B., Wang J., Wei Y., Zhao Y., Jiao Q., Ji J., Yu D., Xu L. (2020). Bioinformatic Analysis Reveals Hub Genes and Pathways That Promote Melanoma Metastasis. BMC Cancer.

[74] Marie K.L., Sassano A., Yang H.H., Michalowski A.M., Michael H.T., Guo T., Tsai Y.C., Weissman A.M., Lee M.P., Jenkins L.M. (2020). Melanoblast Transcriptome Analysis Reveals Pathways Promoting Melanoma Metastasis. Nat. Commun..

[75] Zhang P., Kawakami H., Liu W., Zeng X., Strebhardt K., Tao K., Huang S., Sinicrope F.A. (2018). Targeting CDK1 and MEK/ERK Overcomes Apoptotic Resistance in BRAF-Mutant Human Colorectal Cancer. Mol. Cancer Res..

[76] Anderson N.M., Simon M.C. (2020). The Tumor Microenvironment. Curr. Biol..

[77] Lee N., Zakka L.R., Mihm M.C., Schatton T. (2016). Tumour-Infiltrating Lymphocytes in Melanoma Prognosis and Cancer Immunotherapy. Pathology.

[78] Clemente C.G., Mihm M.C., Bufalino R., Zurrida S., Collini P., Cascinelli N. (1996). Prognostic Value of Tumor Infiltrating Lymphocytes in the Vertical Growth Phase of Primary Cutaneous Melanoma. Cancer.

[79] Mihm M.C., Mulé J.J. (2015). Reflections on the Histopathology of Tumor-Infiltrating Lymphocytes in Melanoma and the Host Immune Response. Cancer Immunol. Res..

[80] Fridman W.H., Zitvogel L., Sautès–Fridman C., Kroemer G. (2017). The Immune Contexture in Cancer Prognosis and Treatment. Nat. Rev. Clin. Oncol..

[81] Long G.V., Hauschild A., Santinami M., Atkinson V., Mandalà M., Chiarion-Sileni V., Larkin J., Nyakas M., Dutriaux C., Haydon A. (2017). Adjuvant Dabrafenib plus Trametinib in Stage III *BRAF*-Mutated Melanoma. N. Engl. J. Med..

[82] Long G.V., Flaherty K.T., Stroyakovskiy D., Gogas H., Levchenko E., de Braud F., Larkin J., Garbe C., Jouary T., Hauschild A. (2017). Dabrafenib plus Trametinib versus Dabrafenib Monotherapy in Patients with Metastatic BRAF V600E/K-Mutant Melanoma: Long-Term Survival and Safety Analysis of a Phase 3 Study. Ann. Oncol. Off. J. Eur. Soc. Med. Oncol..

[83] Hauschild A., Grob J.-J., Demidov L.V., Jouary T., Gutzmer R., Millward M., Rutkowski P., Blank C.U., Miller W.H., Kaempgen E. (2012). Dabrafenib in BRAF-Mutated Metastatic Melanoma: A Multicentre, Open-Label, Phase 3 Randomised Controlled Trial. Lancet.

[84] Sosman J.A., Kim K.B., Schuchter L., Gonzalez R., Pavlick A.C., Weber J.S., McArthur G.A., Hutson T.E., Moschos S.J., Flaherty K.T. (2012). Survival in BRAF V600–Mutant Advanced Melanoma Treated with Vemurafenib. N. Engl. J. Med..

[85] Long G.V., Stroyakovskiy D., Gogas H., Levchenko E., de Braud F., Larkin J., Garbe C., Jouary T., Hauschild A., Grob J.J. (2014). Combined BRAF and MEK Inhibition versus BRAF Inhibition Alone in Melanoma. N. Engl. J. Med..

[86] Pires da Silva I., Wang K.Y.X., Wilmott J.S., Holst J., Carlino M.S., Park J.J., Quek C., Wongchenko M., Yan Y., Mann G. (2019). Distinct Molecular Profiles and Immunotherapy Treatment Outcomes of V600E and V600K *BRAF*-Mutant Melanoma. Clin. Cancer Res..

[87] McArthur G.A., Chapman P.B., Robert C., Larkin J., Haanen J.B., Dummer R., Ribas A., Hogg D., Hamid O., Ascierto P.A. (2014). Safety and Efficacy of Vemurafenib in BRAF(V600E) and BRAF(V600K) Mutation-Positive Melanoma (BRIM-3): Extended Follow-up of a Phase 3, Randomised, Open-Label Study. Lancet Oncol..

[88] Ascierto P.A., Minor D., Ribas A., Lebbe C., O’Hagan A., Arya N., Guckert M., Schadendorf D., Kefford R.F., Grob J.-J. (2013). Phase II Trial (BREAK-2) of the BRAF Inhibitor Dabrafenib (GSK2118436) in Patients with Metastatic Melanoma. J. Clin. Oncol..

[89] Simanshu D.K., Nissley D.V., McCormick F. (2017). RAS Proteins and Their Regulators in Human Disease. Cell.

[90] Ascierto P.A., Schadendorf D., Berking C., Agarwala S.S., van Herpen C.M., Queirolo P., Blank C.U., Hauschild A., Beck J.T., St-Pierre A. (2013). MEK162 for Patients with Advanced Melanoma Harbouring NRAS or Val600 BRAF Mutations: A Non-Randomised, Open-Label Phase 2 Study. Lancet Oncol..

[91] Dummer R., Schadendorf D., Ascierto P.A., Arance A., Dutriaux C., Di Giacomo A.M., Rutkowski P., Del Vecchio M., Gutzmer R., Mandala M. (2017). Binimetinib versus Dacarbazine in Patients with Advanced NRAS-Mutant Melanoma (NEMO): A Multicentre, Open-Label, Randomised, Phase 3 Trial. Lancet Oncol..

[92] Lebbé C., Dutriaux C., Lesimple T., Kruit W., Kerger J., Thomas L., Guillot B., de Braud F., Garbe C., Grob J.-J. (2020). Pimasertib Versus Dacarbazine in Patients with Unresectable NRAS-Mutated Cutaneous Melanoma: Phase II, Randomized, Controlled Trial with Crossover. Cancers.

[93] Cox A.D., Der C.J., Philips M.R. (2015). Targeting RAS Membrane Association: Back to the Future for Anti-RAS Drug Discovery?. Clin. Cancer Res..

[94] Niessner H., Beck D., Sinnberg T., Lasithiotakis K., Maczey E., Gogel J., Venturelli S., Berger A., Mauthe M., Toulany M. (2011). The Farnesyl Transferase Inhibitor Lonafarnib Inhibits MTOR Signaling and Enforces Sorafenib-Induced Apoptosis in Melanoma Cells. J. Investig. Dermatol..

[95] Randic T., Kozar I., Margue C., Utikal J., Kreis S. (2021). NRAS Mutant Melanoma: Towards Better Therapies. Cancer Treat. Rev..

[96] Ryan M.B., Corcoran R.B. (2018). Therapeutic Strategies to Target RAS-Mutant Cancers. Nat. Rev. Clin. Oncol..

[97] Carvajal R.D. (2011). KIT as a Therapeutic Target in Metastatic Melanoma. JAMA.

[98] Hodi F.S., Corless C.L., Giobbie-Hurder A., Fletcher J.A., Zhu M., Marino-Enriquez A., Friedlander P., Gonzalez R., Weber J.S., Gajewski T.F. (2013). Imatinib for Melanomas Harboring Mutationally Activated or Amplified *KIT* Arising on Mucosal, Acral, and Chronically Sun-Damaged Skin. J. Clin. Oncol..

[99] Guo J., Carvajal R.D., Dummer R., Hauschild A., Daud A., Bastian B.C., Markovic S.N., Queirolo P., Arance A., Berking C. (2017). Efficacy and Safety of Nilotinib in Patients with KIT-Mutated Metastatic or Inoperable Melanoma: Final Results from the Global, Single-Arm, Phase II TEAM Trial. Ann. Oncol..

[100] Janku F., Bauer S., Shoumariyeh K., Jones R.L., Spreafico A., Jennings J., Psoinos C., Meade J., Ruiz-Soto R., Chi P. (2022). Efficacy and Safety of Ripretinib in Patients with KIT-Altered Metastatic Melanoma. ESMO Open.

[101] Hofmann U.B., Kauczok-Vetter C.S., Houben R., Becker J.C. (2009). Overexpression of the KIT/SCF in Uveal Melanoma Does Not Translate into Clinical Efficacy of Imatinib Mesylate. Clin. Cancer Res..

[102] Calipel A., Landreville S., De La Fouchardière A., Mascarelli F., Rivoire M., Penel N., Mouriaux F. (2014). Mechanisms of Resistance to Imatinib Mesylate in KIT-Positive Metastatic Uveal Melanoma. Clin. Exp. Metastasis.

[103] Garraway L.A., Widlund H.R., Rubin M.A., Getz G., Berger A.J., Ramaswamy S., Beroukhim R., Milner D.A., Granter S.R., Du J. (2005). Integrative Genomic Analyses Identify MITF as a Lineage Survival Oncogene Amplified in Malignant Melanoma. Nature.

[104] Hoek K.S., Schlegel N.C., Brafford P., Sucker A., Ugurel S., Kumar R., Weber B.L., Nathanson K.L., Phillips D.J., Herlyn M. (2006). Metastatic Potential of Melanomas Defined by Specific Gene Expression Profiles with No BRAF Signature. Pigment Cell Res..

[105] Tirosh I., Izar B., Prakadan S.M., Wadsworth M.H., Treacy D., Trombetta J.J., Rotem A., Rodman C., Lian C., Murphy G. (2016). Dissecting the Multicellular Ecosystem of Metastatic Melanoma by Single-Cell RNA-Seq. Science.

[106] Hugdahl E., Kalvenes M.B., Mannelqvist M., Ladstein R.G., Akslen L.A. (2018). Prognostic Impact and Concordance of TERT Promoter Mutation and Protein Expression in Matched Primary and Metastatic Cutaneous Melanoma. Br. J. Cancer.

[107] Diaz A., Puig-Butillé J.A., Muñoz C., Costa D., Díez A., Garcia-Herrera A., Carrera C., Badenas C., Solé F., Malvehy J. (2014). TERT Gene Amplification Is Associated with Poor Outcome in Acral Lentiginous Melanoma. J. Am. Acad. Dermatol..

[108] Del Bianco P., Stagni C., Giunco S., Fabozzi A., Elefanti L., Pellegrini S., Vecchiato A., Pigozzo J., Zamuner C., De Rossi A. (2020). TERT Promoter Mutations Differently Correlate with the Clinical Outcome of MAPK Inhibitor-Treated Melanoma Patients. Cancers.

[109] Reyes-Uribe P., Adrianzen-Ruesta M.P., Deng Z., Echevarria-Vargas I., Mender I., Saheb S., Liu Q., Altieri D.C., Murphy M.E., Shay J.W. (2018). Exploiting TERT Dependency as a Therapeutic Strategy for NRAS-Mutant Melanoma. Oncogene.

[110] Li H., Li J., Zhang C., Zhang C., Wang H. (2020). TERT Mutations Correlate with Higher TMB Value and Unique Tumor Microenvironment and May Be a Potential Biomarker for Anti-CTLA4 Treatment. Cancer Med..

[111] Lee B., Sandhu S., McArthur G. (2015). Cell Cycle Control as a Promising Target in Melanoma. Curr. Opin. Oncol..

[112] Mao L., Dai J., Cao Y., Bai X., Sheng X., Chi Z., Cui C., Kong Y., Zhang Y., Wu L. (2021). Palbociclib in Advanced Acral Melanoma with Genetic Aberrations in the Cyclin-Dependent Kinase 4 Pathway. Eur. J. Cancer.

[113] Garutti M., Targato G., Buriolla S., Palmero L., Minisini A.M., Puglisi F. (2021). CDK4/6 Inhibitors in Melanoma: A Comprehensive Review. Cells.

[114] Kwong L.N., Costello J.C., Liu H., Jiang S., Helms T.L., Langsdorf A.E., Jakubosky D., Genovese G., Muller F.L., Jeong J.H. (2012). Oncogenic NRAS Signaling Differentially Regulates Survival and Proliferation in Melanoma. Nat. Med..

[115] Schuler M., Zimmer L., Kim K.B., Sosman J.A., Ascierto P.A., Postow M.A., De Vos F.Y.F.L., van Herpen C.M.L., Carlino M.S., Johnson D.B. (2022). Phase Ib/II Trial of Ribociclib in Combination with Binimetinib in Patients with *NRAS*-Mutant Melanoma. Clin. Cancer Res..

[116] Hodi F.S., O’Day S.J., McDermott D.F., Weber R.W., Sosman J.A., Haanen J.B., Gonzalez R., Robert C., Schadendorf D., Hassel J.C. (2010). Improved Survival with Ipilimumab in Patients with Metastatic Melanoma. N. Engl. J. Med..

[117] Arheden A., Skalenius J., Bjursten S., Stierner U., Ny L., Levin M., Jespersen H. (2019). Real-World Data on PD-1 Inhibitor Therapy in Metastatic Melanoma. Acta Oncol..

[118] Hamid O., Robert C., Daud A., Hodi F.S., Hwu W.J., Kefford R., Wolchok J.D., Hersey P., Joseph R., Weber J.S. (2019). Five-Year Survival Outcomes for Patients with Advanced Melanoma Treated with Pembrolizumab in KEYNOTE-001. Ann. Oncol..

[119] Hodi F.S., Chiarion-Sileni V., Gonzalez R., Grob J.-J., Rutkowski P., Cowey C.L., Lao C.D., Schadendorf D., Wagstaff J., Dummer R. (2018). Nivolumab plus Ipilimumab or Nivolumab Alone versus Ipilimumab Alone in Advanced Melanoma (CheckMate 067): 4-Year Outcomes of a Multicentre, Randomised, Phase 3 Trial. Lancet Oncol..

[120] Schadendorf D., Hodi F.S., Robert C., Weber J.S., Margolin K., Hamid O., Patt D., Chen T.-T., Berman D.M., Wolchok J.D. (2015). Pooled Analysis of Long-Term Survival Data from Phase II and Phase III Trials of Ipilimumab in Unresectable or Metastatic Melanoma. J. Clin. Oncol..

[121] Brahmer J.R., Tykodi S.S., Chow L.Q.M., Hwu W.-J., Topalian S.L., Hwu P., Drake C.G., Camacho L.H., Kauh J., Odunsi K. (2012). Safety and Activity of Anti-PD-L1 Antibody in Patients with Advanced Cancer. N. Engl. J. Med..

[122] Hamid O., Robert C., Daud A., Hodi F.S., Hwu W.-J., Kefford R., Wolchok J.D., Hersey P., Joseph R.W., Weber J.S. (2013). Safety and Tumor Responses with Lambrolizumab (Anti-PD-1) in Melanoma. N. Engl. J. Med..

[123] Topalian S.L., Hodi F.S., Brahmer J.R., Gettinger S.N., Smith D.C., McDermott D.F., Powderly J.D., Carvajal R.D., Sosman J.A., Atkins M.B. (2012). Safety, Activity, and Immune Correlates of Anti-PD-1 Antibody in Cancer. N. Engl. J. Med..

[124] Goodman A.M., Kato S., Bazhenova L., Patel S.P., Frampton G.M., Miller V., Stephens P.J., Daniels G.A., Kurzrock R. (2017). Tumor Mutational Burden as an Independent Predictor of Response to Immunotherapy in Diverse Cancers. Mol. Cancer Ther..

[125] Patterson A., Auslander N. (2022). Mutated Processes Predict Immune Checkpoint Inhibitor Therapy Benefit in Metastatic Melanoma. Nat. Commun..

[126] Gandini S., Massi D., Mandalà M. (2016). PD-L1 Expression in Cancer Patients Receiving Anti PD-1/PD-L1 Antibodies: A Systematic Review and Meta-Analysis. Crit. Rev. Oncol. Hematol..

[127] Kaunitz G.J., Cottrell T.R., Lilo M., Muthappan V., Esandrio J., Berry S., Xu H., Ogurtsova A., Anders R.A., Fischer A.H. (2017). Melanoma Subtypes Demonstrate Distinct PD-L1 Expression Profiles. Lab. Investig..

[128] Danilova L., Wang H., Sunshine J., Kaunitz G.J., Cottrell T.R., Xu H., Esandrio J., Anders R.A., Cope L., Pardoll D.M. (2016). Association of PD-1/PD-L Axis Expression with Cytolytic Activity, Mutational Load, and Prognosis in Melanoma and Other Solid Tumors. Proc. Natl. Acad. Sci. USA.

